# Three new species of *Solanum* (Brevantherum Clade) endemic to the Brazilian Atlantic Forest

**DOI:** 10.3897/phytokeys.38.7055

**Published:** 2014-06-04

**Authors:** Leandro L. Giacomin, João R. Stehmann

**Affiliations:** 1 ICB, Departamento de Botânica, Laboratório de Sistemática Vegetal, Universidade Federal de Minas Gerais – UFMG, Av. Antônio Carlos, 6627, Pampulha, Belo Horizonte, CEP 31270-901, MG, Brazil

**Keywords:** Atlantic Forest, Brazil, Brevantherum Clade, IUCN conservation status, *Solanum*

## Abstract

Three new Brazilian species of the Brevantherum clade of *Solanum* (Solanaceae) are described, all closely related to the poorly known *Solanum inornatum* Witasek. *Solanum bradei* Giacomin & Stehmann, **sp. nov.,** and *Solanum kriegeri* Giacomin & Stehmann, **sp. nov.,** differ from *S. inornatum* in having very small deltate calyx lobes that are not accrescent in fruit. *Solanum bradei* is a shrub up to 1.8 m with generally pedunculate inflorescences and tiny translucent fruits, whereas *Solanum kriegeri* is a dwarf glabrescent plant growing on sandy soils in cloud forests, with larger fruits and sessile to subsessile inflorescence. *Solanum friburgense* Giacomin & Stehmann, **sp. nov.,** has linear calyx lobes like *S. inornatum*, and is characterized by its 2-foliate sympodia and leaf pubescence, with trichomes concentrated on leaf veins. The species here described and illustrated are restricted to the mountain ranges of Mantiqueira and Serra do Mar in the Atlantic forests of southeastern Brazil and are all of considerable conservation concern.

## Abstract (pt)

Três novas espécies pertencentes ao clado Brevantherum do gênero *Solanum* (Solanaceae) são descritas, todas proximamente relacionadas a *Solanum inornatum* Witasek. *Solanum bradei* Giacomin & Stehmann, **sp. nov.,** e *Solanum kriegeri* Giacomin & Stehmann, **sp. nov.,** diferem de *S. inornatum* por apresentarem lobos do cálice deltoides, muito pequenos, não acrescentes no fruto, mas a primeira se destaca por apresentar um porte marcadamente maior, chegando a formar arbustos de até 1.8 m, com inflorescências geralmente pedunculadas e frutos diminutos translúcidos, enquanto *Solanum kriegeri* é uma forma anã e glabrescente que habita matas nebulares associadas a solos arenosos. *Solanum friburgense* Giacomin & Stehmann, **sp. nov.,** apresenta lobos do cálice lineares, como em *S. inornatum*, mas se diferencia pelo simpódio 2-foliado e indumento das folhas, com tricomas concentrados nas nervuras. As espécies aqui descritas e ilustradas são restritas às Serras da Mantiqueira e do Mar no domínio Atlântico do sudeste brasileiro e são indicadas como ameaçadas de extinção.

## Introduction

The phytogeographic domain of the Atlantic Forest in South America is a complex of ecosystems and is recognized as one of the most biodiverse regions on earth (Mittermeier et al. 2004). Most of its extent lies in Brazilian territory (up to 95%), but it also reaches Argentina and Paraguay and is estimated to originally comprise an area between 1,300,000 and 1,500,000 square kilometers (about 15% of Brazilian territory; Morellato and Haddad 2000). The main urban centers in Brazil are within the Atlantic Forest domain, and their cycles of expansion and occupation have led to critical levels of reduction in these originally forested environments (Dean 1996). It is estimated that only 12 to 16% of the original forest cover remains, mostly in small fragments (Ribeiro et al. 2009). Despite this reduction, discovery of new species in the Atlantic forests is still ongoing and reflects a still insufficiently known diversity. For example, 42% of the newly described angiosperm species for Brazil between 1990–2006 were based on collections from this phytogeographic domain (Sobral and Stehmann 2009).

Brazil contains more than a fifth of the species of Solanaceae, mainly due to the richness found in the Atlantic Forest (Stehmann et al. 2009, Stehmann et al. 2013), making this one of the centers of diversity of the family (Knapp 2002a). More than half of Solanaceae species are members of the giant genus *Solanum* L., a proportion also observed in the Brazilian flora. This genus includes globally important crops such as the potato (*Solanum tuberosum* L.) and the tomato (*Solanum lycopersicum* L.). Of the 266 species of *Solanum* listed in Brazil, 68% (181) of them are known to occur in the Atlantic Forest and 46% (124) are restricted to the domain (Stehmann et al. 2013). Ongoing inventories of the Atlantic Forest and work on a preliminary Solanaceae flora of the country has revealed many new species and new distributional records for poorly known taxa; here we describe three of these new species.

The species here described are all closely related to *Solanum inornatum* Witasek, a narrowly distributed and poorly known species from São Paulo state. It has not yet been assigned to any formal infrageneric division of *Solanum*, but was thought in the past to be related to section *Gonatotrichum* Bitter due to a similar habit and hair morphology (L.A. Mentz, pers. comm.). Recent phylogenetic studies using molecular characters (Giacomin 2010, L.L. Giacomin et al. in prep.) showed the *Solanum inornatum* species group (defined here as including *Solanum inornatum* and the three species described here) is part of the larger Brevantherum Clade (*sensu* Weese and Bohs 2007, Särkinen et al. 2013), one of the 12 to 15 main lineages of *Solanum*. The *Solanum inornatum* group is part of a well-supported monophyletic group, together with all other species of the Brevantherum clade, that is sister to a clade formed by members of section *Gonatotrichum* (Giacomin 2010, L.L. Giacomin et al. in prep.).

Species of the Brevantherum Clade are native to the New World, occurring from southern United States through Argentina, with a center of diversity in the Brazilian Atlantic Forest. A few species are widespread and invasive in tropical regions in the Old World (e.g., *Solanum erianthum* D. Don, *Solanum mauritianum* Scop.). Morphologically, members of the Brevantherum Clade are unarmed herbs to shrubs or small trees with unbranched (section *Gonatotrichum* and members of *Solanum inornatum* species group) to variously branched or stellate trichomes (all other species), and small oblong-ellipsoid poricidal anthers with introrsely opening pores. The species of the *Solanum inornatum* group are herbs to small shrubs with exclusively unbranched trichomes and are all restricted to southeastern Brazil. Although they share unbranched (and rarely geniculate; see Giacomin 2010, Stern et al. 2013) trichomes with section *Gonatotrichum*, members of *Solanum inornatum* group are morphologically and phylogenetically distinct from those species. They can be readily distinguished from the species of section *Gonatotrichum* in that they lack the characteristic explosive fruit dehiscence (Stern et al. 2013) and have more deeply lobed corollas. All the other species of Brevantherum clade except the *Solanum inornatum* group and section *Gonatotrichum* are shrubs to trees with stellate or lepidote trichomes (Giacomin 2010). A revision of the morphological delimitation of the *Solanum inornatum* species group as well as a discussion of its phylogenetic position will be subject of another manuscript that is in preparation (L.L. Giacomin et al. in prep.).

## Materials and methods

Material from the following herbaria were studied (acronyms from *Index Herbariorum*, http://sweetgum.nybg.org/ih/ ): BHCB, BM, CESJ, ESA, FUEL, MBM, MBML, R, RB, SP, SPF, SPSF, UEC, UPCB, VIC, W and WU. Cited material is ordered geographically. Barcodes of type specimens, when present, are noted in square brackets after the herbarium citation. Plants obtained in the field were cultivated in a greenhouse in Belo Horizonte and fresh flowers were fixed in alcohol to permit detailed descriptions and illustrations. We assessed the conservation status using IUCN Red List Categories and Criteria (IUCN 2013). For the estimation of the extent of occurrence (EOO) and area of occupancy (AOO) we used the CAT tool described in Bachman et al. (2011) and available at http://geocat.kew.org/ . For the AOO estimation was used the standard IUCN cell size of 4 km^2^.

## Taxonomic treatment

### Solanum bradei

Giacomin & Stehmann sp. nov.

urn:lsid:ipni.org:names:77139681-1

http://species-id.net/wiki/Solanum_bradei

Figs 1 2A, B

#### Diagnosis.

Differs from all other species of the *Solanum inornatum* group in its shrub-like, woody habit and long-pedunculate inflorescences (peduncles up to 1 cm). Unlike *Solanum inornatum* Witasek it has deltate, rather than linear-lanceolate, calyx lobes that are not accrescent in fruit.

#### Type.

BRAZIL. Rio de Janeiro: Mun. Itatiaia. Parque Nacional do Itatiaia, continuação da BR após posto de vigilância, margens da estrada próximo a casa, 1171 m, 22°26'11.15"S, 44°37'27.55"W, 3 Nov 2008 (fl), *L.L. Giacomin, L.H.Y. Kamino & T.E. Almeida 359* (holotype: BHCB [BHCB-012523]; isotypes: BM, NY, RB).

#### Description.

Herbs to shrubs, woody at base, erect, to 1.8 m tall, usually much-branched, the upper branches decumbent, flexuous on young plants. Stems moderate to densely pubescent with simple uniseriate 2–3(4)-celled trichomes up to 2.8 mm long, these appressed, arcuate, or erect along stems, or sometimes geniculate and antrorse. Bark of older stems turning brownish-green, glabrescent, not exfoliating, normally matte brown on new growth. Sympodial units 3-plurifoliate, mostly geminate, when so markedly anisophyllous, differing in shape and size, with the smaller leaves highly reduced. Leaves simple, the major ones 2–11.5 × 0.9–3 cm, lanceolate to narrowly-elliptic, chartaceous, slightly discolorous, drying light green beneath, dark green above, not shiny, moderate to densely pubescent in both surfaces with unbranched antrorse, appressed, or erect hairs, with up to 3 cells; base attenuate to rounded, sometimes slightly asymmetric, not decurrent onto the petiole; margins entire, sometimes slightly revolute, ciliate, with the trichomes lying antrorsely parallel to the margin; apex acute to acuminate; petioles 2–13 mm long, with pubescence similar to the stems; minor ones 0.35–2.6 × 0.2–1.3 cm, broadly elliptic to circular; the base obtuse to rounded, margins like those of the major leaves; the apex rounded to acute, the petioles absent to 3 mm; venation brochidodromous; midrib and secondary veins visible to the naked eye, prominent abaxially, with only the midrib slightly prominent to impressed adaxially. Inflorescences pedunculate, terminal, lateral or sub-opposite the leaves, unbranched cymes with 3–7 flowers, the axis with pubescence like that of the stems; peduncle 2.2–10 mm long; pedicels 2–8 mm in the flower, 6–14 mm in fruit, articulated at base, spaced up to 8 mm apart. Calyx up to 3 mm long, the lobes 1–2 mm long in flower and fruit, about 1 mm wide, the lobes ovate-deltate, with an acuminate apex, abaxially moderately to densely pubescent with antrorse trichomes like those of the stem abaxially, adaxially densely pubescent with capitate glandular trichomes less than 1 mm long, with single-celled stalks and a multicellular head; calyx not accrescent in fruit. Corolla 6–10 mm in diameter, white, stellate, membranaceous, the lobes 3–5 × 2–3 mm, ovate-lanceolate, glabrous abaxially and adaxially. Stamens 2–3 mm long, equal in length, the filaments ca. 1 mm long; anthers 1–2 mm long, ca. 1 mm wide, oblong-ellipsoid, slightly connivent, yellow, the base cordate, the apex emarginate and poricidal, the subapical pores directed introrsely, not opening into longitudinal slits. Ovary glabrous; style white, 4–6 mm long, exserted beyond stamens, straight, cylindrical, glabrous, the stigma light green, capitate. Fruit a globose berry 4–7.8 mm in diameter, slightly translucent green to dull green when ripe, drying dark, glabrous. Seeds 2–4 per berry, 2–4.5 × 2–3.3 mm, flattened, ovate-reniform, with a small hollow at hilum region; the seed surface undulate; margins flattened.

**Figure 1.**
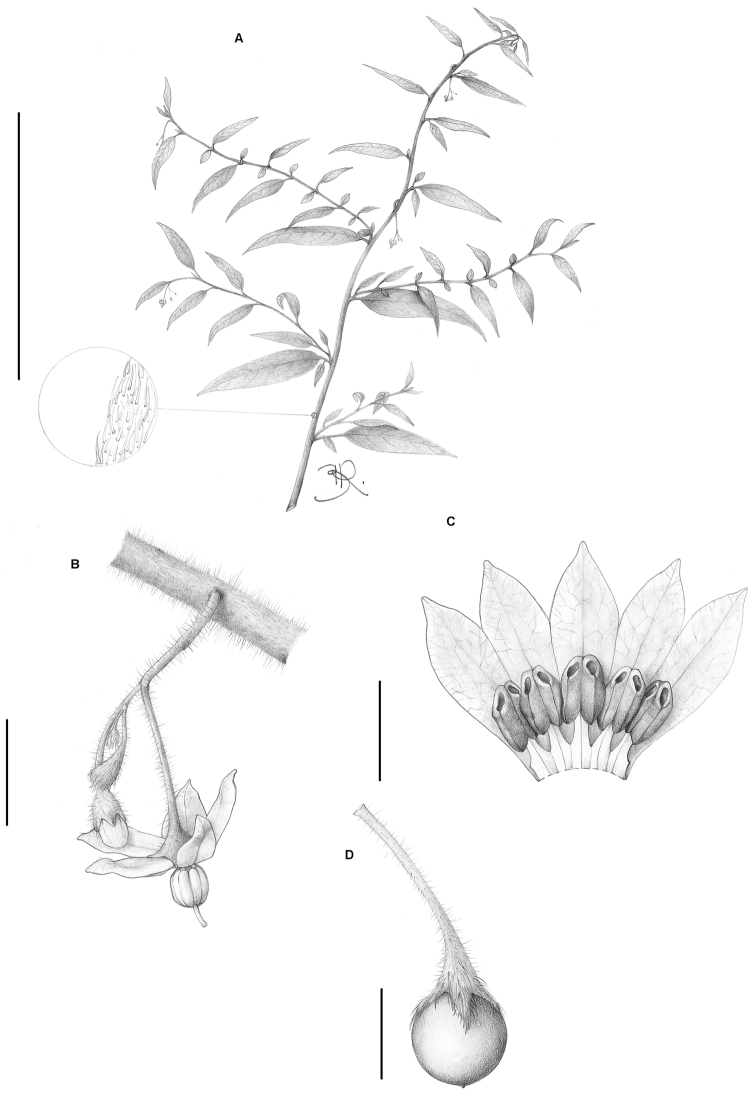
*Solanum bradei* Giacomin & Stehmann. **A** Habit **B** Inflorescence detail, showing the long peduncle **C** Corolla cross section showing stamens **D** Fruit, note the small, deltate calyx lobes. All from *Giacomin et al. 359* (BHCB). Scale bars **A**= 5 cm; **B** and **D** = 5 mm; **C** = 2 mm. Drawings by B. Raddichi.

**Figure 2.**
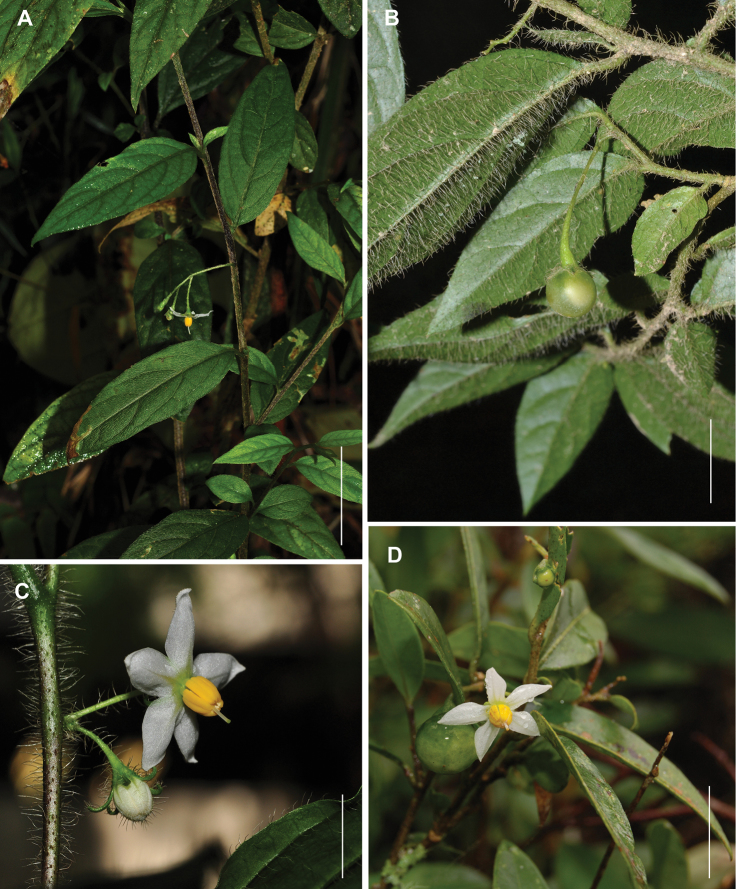
Photographs of the species in the field. **A–B** *Solanum bradei* Giacomin & Stehmann **A** Shoot showing the anisophyllous sympodia and lateral emergence of the inflorescence [from *Hattori et al. 914* (BHCB)] **B** Translucent fruit [from *Agra et al. 7398* (BHCB, JPB)] **C** Inflorescence of *Solanum friburgense* Giacomin & Stehmann in cultivation showing its hispid indumentum [from *Giacomin 940* (BHCB)] **D** Inflorescence of *Solanum kriegeri* Giacomin & Stehmann [from *Giacomin et al*. 770 (BHCB); photo by F.D. Gontijo] . Scale bars **A**= 2 cm; **B–D**= 1 cm.

#### Distribution.

**Figure 3.**
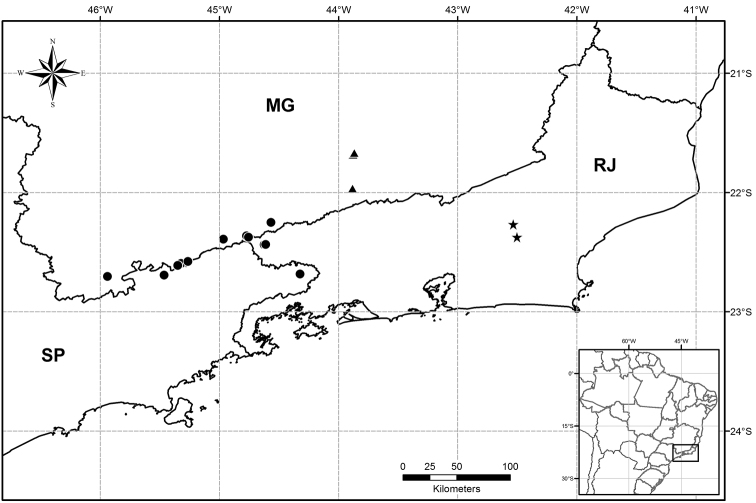
Known distribution of *Solanum bradei* Giacomin & Stehmann (circles), *Solanum friburgense* Giacomin & Stehmann (stars) and *Solanum kriegeri* Giacomin & Stehmann (triangles) in the states of Minas Gerais (**MG**), Rio de Janeiro (**RJ**) and São Paulo (**SP**) in southeastern Brazil.

Restricted to the Brazilian states of Minas Gerais, Rio de Janeiro and São Paulo (Fig. 3). The known specimens are mainly from the Mantiqueira mountain range in the border area between those states, with one disjunct collection from Serra do Mar, in the Bocaina region of northeastern São Paulo State.

#### Ecology.

Occasional in the understory or shaded forest edges of well-preserved or secondary fragments of the Brazilian Atlantic coastal rainforest (*Floresta Ombrófila Densa* of Veloso et al. 1991), normally close to water courses, in elevations ranging from 1,000 to 2,000 m. In cultivation in Belo Horizonte, *Solanum bradei* flowered year round. Preliminary crossing studies suggested it was self-incompatible as no fruits were produced in selfed plants, but more individuals should be used for a definitive conclusion.

#### Phenology.

*Solanum bradei* seems to produce flowers year round but a flowering peak is observed in the rainy season, as most of the collections are from between the months of October and March. None of the examined collections are from December or January. Most of the specimens flowering in October and November and fruiting from February through May. The only well developed fruit was found in a collection from May.

#### Etymology.

The epithet honors the German botanist Alexander C. Brade [1881–1971] who greatly contributed to the growth of botany in Brazil in the middle 1900s. His efforts to better understand the flora of Itatiaia led to the earliest known collections of *Solanum bradei*.

#### Preliminary conservation status

**(IUCN, 2013).** Endangered (EN) B1; B2 ab (ii,iii,iv). The EOO and AOO calculated were 4,076.04 km^2^ and 48 km^2^ respectively resulting in the assessment of the Endangered category. The species is known from eight localities only, most of which are subject to urban expansion and deforestation due to tourism and agriculture. Although the species is known to occur in three protected areas [Área de Preservação Ambiental Serra da Mantiqueira (APA Mantiqueira), Parque Estadual de Campos do Jordão and Parque Nacional de Itatiaia] we suggest to maintain it as Endangered due to: the effectiveness of APAs in protecting the species is doubtful, the Parque Estadual de Campos do Jordão have considerable areas with exotic species, and both it and Parque Nacional do Itatiaia have considerable areas with habitats not suitable to *Solanum bradei* (such as outcrops and highland grassfields). Although *Solanum bradei* is known to grow on secondary fragments and in a wide elevation range, threats to it are clear, considering that the southern Mantiqueira range, where most collections are from, is situated between the two main urban centers in Brazil and has become a tourism hub. In addition, over the past few decades the montane forests and the highland fields of Mantiqueira have been increasingly converted to pastures, monocultures or urban centers.

#### Discussion.

*Solanum bradei* is the most widely distributed and morphologically variable species of the *Solanum inornatum* species group. It is the only species of the group not necessarily associated with well-preserved sites, although always found in shaded environments, and is also the one that has the broadest elevational range. Despite the usual shrubby, robust habit of the species, specimens as small as 20–25 cm tall were found flowering, these mainly from the municipalities of Camanducaia and Gonçalves, in southern Minas Gerais State. The species has distinctive geminate sympodia, with leaves differing in shape and size to a degree not observed in any other species of the group. Although this character was observed in all specimens in the field, some branches preserved on herbarium sheets do not retain well-developed minor leaves. Due to this, anisophylly was not used as a diagnostic character to separate the species, but it is certainly a useful character in the field.

Some plants seem to develop diseased flowers, possibly the result of fungal or viral infection, as has been previously reported in other species of *Solanum* (see Hernández and Hennen 2003). In these cases an unusual form of calyx growth is observed where the expanded calyx covers the entire flower, making it resemble a fruit. This has resulted in misleading annotations on some labels [e.g. *Polisel et al. 228* (SPSF) has “Fruto imaturo verde” written on the label but the specimen is actually flowering]. When dissected, the diseased flowers show an opened and lobed corolla, retained in the expanded calyx, and purplish blue anthers (in dried material) that produce less pollen than normal. These putatively infertile flowers are more common in young plants from disturbed areas.

*Solanum bradei* can be easily distinguished from *Solanum inornatum* by its tiny deltate calyx lobes (1–2 mm long) that are not accrescent in fruit and its long-pedunculate inflorescence (peduncles up to 1 cm). *Solanum inornatum* has linear, 3–5 mm long calyx lobes and inflorescences with peduncles 1–4 mm long. The characters that separate it from the other species described in this paper are discussed further below, on each species discussion.

In the past, sheets of *Solanum bradei* have been determined as *Solanum apiahyense* Witasek by various *Solanum* taxonomists, another poorly known species of uncertain affinities from secondary formations in São Paulo, Paraná and Santa Catarina states. More recently, *Solanum apiahyense* was found to be closely related to *Solanum trachytrichium* Bitter, a species previously assigned to the Geminata clade (Knapp 2002b, L.L. Giacomin in prep.). Among species that could be confused with members of *Solanum inornatum* group and that are not considered part of the Brevantherum clade, these two (*Solanum apiahyense* and *Solanum trachytrichium*) are the only ones that we judge should be mentioned here. They can be distinguished from all species of the *Solanum inornatum* group by having inflorescences with fruiting peduncle longer than 1 cm and pedicels apically expanded with a constriction at the receptacle. The pubescence of the species are also distinctive: while *Solanum apiahyense* has very long, multicellular (up to 7 cells) unbranched trichomes, *Solanum trachytrichium* has unicellular trichomes that are hooked on a mound-like base, giving the leaves an scabrous aspect, rough to the touch (Knapp 2002b).

#### Specimens examined.

**BRAZIL**. **Minas Gerais**: **Mun. Camanducaia**. Próximo a Gonçalves, Na mata do Sr. Altair, 1900 m, 23 Oct 2001 (fl), *J.R. Stehmann & I.B. Castro 3022* (BHCB, RB); Mun. Camanducaia. Divisa com Gonçalves, Próximo a Pedra de São Domingos, 1727 m, 22°42'27.1"S, 45°56'1.39"W, 12 Mar 2003 (fl, fr), *J.R. Stehmann & G.S. França 3415* (BHCB); same locality, 12 Mar 2003 (fl, fr), *J.R. Stehmann & G.S. França 3416* (BHCB); same locality 12 Mar 2003 (fl, fr), *J.R. Stehmann & G.S. França 3417* (BHCB). **Mun. Delfim Moreira**. Margens da estrada que liga Delfim Moreira a Campos do Jordão, 1781 m, 22°34'40.84"S, 45°15'49.07"W, 1 Nov 2008 (fl), *L.L. Giacomin et al. 319* (BHCB); same locality, 1673 m, 22°35'40.93"S, 45°19'19.81"W, 1 Nov 2008 (fl), *L.L. Giacomin et al. 346* (BHCB); Fazenda da Onça (área mantida pelo Exército Brasileiro), próximo ao pórtico de entrada da fazenda, 1674 m, 22°36'41"S, 45°20'56"W, 15 Mar 2011 (fl), *L.L. Giacomin et al. 1372* (BHCB). **Mun. Gonçalves.** Às margens da estrada de terra que liga Gonçalves a BR-381, Próximo a mata do Altair, 1786 m, 22°42'15.33"S, 45°56'20.16"W, 13 Jul 2008 (bs), *L.L. Giacomin & J.R. Stehmann 180* (BHCB); same locality, 1786 m, 22°42'13,57"S, 45°56'18,59"W, 28 Oct 2008 (fl), *L.L. Giacomin et al. 257* (BHCB). **Mun. Itamonte.** [close to] Parque Nacional de Itatiaia, Outskirts of park on road toward Agulhas Negras, 1728 m, 22°22'25", 44°45'17", 6 May 2011 (fr), *M.F. Agra et al. 7398* (BHCB, JPB); Margens da BR-354, 1558 m, 22°22'25", 44°45'17"22°21'47.10"S, 44°46'23.05"W, 12 Jul 2008 (fl), *L.L. Giacomin & J.R. Stehmann 171* (BHCB); same locality, 1558 m, 22°21'45.17"S, 44°46'21.52"W, 5 Nov 2008 (fl), *L.L. Giacomin et al. 372* (BHCB); Margens da rodovia não pavimentada que leva para o Pico das Agulhas Negras e parte alta do Parque Nacional do Itatiaia (BR-485); ca. 1 km após a entrada, 1711 m, 22°22'24"S, 44°45'19"W, 20 Nov 2013 (fl), *L.L. Giacomin et al. 2028* (BHCB, BM, UT); Estrada para Rio de Janeiro, 1576 m, 22°21'49.13"S, 44°46'26.67"W, 22 Nov 2006 (fl, fr), *J.R. Stehmann et al. 4503* (BHCB; ESA). **Mun. Passa Quatro**. Serra da Mantiqueira, Fazenda São Bento, 1700 m, Nov 1948 (bs, fr), *J. Vidal s.n*. (R 209896). **Rio de Janeiro**: **Mun. Itatiaia**. Maromba, Arbusto nas pedras, 3 Feb 1945 (fl), *A.C. Brade 17391* (BHCB, RB); Caminho Rio Bonito, 2 Feb 1948 (fl, fr), *A.C. Brade 18802* (RB); Parque Nacional do Itatiaia, Trilha do Hotel Simon para o Três Picos, 1500 m, 22°15'S, 44°34'W, 23 Nov 1994 (fl), *J.M.A. Braga 1629* (RB); Parque Nacional do Itatiaia, próximo a cachoeira da Maromba, 1186 m, 22°26'10.43"S, 44°37'29.14"W, 3 Nov 2008 (fl, fr), *L.L. Giacomin et al. 357* (BHCB); Parque Nacional do Itatiaia, Trilha do Hotel Simon para o Três Picos, 1087 m, 22°26'7.5"S, 44°36'38.14"W, 3 Nov 2008 (fl), *L.L. Giacomin et al. 361* (BHCB); Parque Nacional do Itatiaia, Trilha do Véu da Noiva, 1163 m, 22°26'4"S, 44°37'24"W, 16 Oct 2009 (fl), *E.K.O. Hattori et al. 914* (BHCB); Parque Nacional do Itatiaia, Trilha para o Rebouças, 16 Oct 2009 (fl), *E.K.O. Hattori et al*. 927 (BHCB). **São Paulo**: **Mun. Bananal.** Serra da Bocaina, Sertão do Rio Vermelho, 1200 m, 6 Oct 1949 (fl), *A.C. Brade & A.Duarte 20106* (RB). **Mun. Campos do Jordão.** Parque Estadual de Campos do Jordão, Trilha da cachoeira, 22°41'30"S, 45°27'52"W, 27 Apr 2007 (bs, fl), *R.T. Polisel et al. 228* (SPSF).

### Solanum friburgense

Giacomin & Stehmann sp. nov.

urn:lsid:ipni.org:names:77139689-1

http://species-id.net/wiki/Solanum_friburgense

Figs 2C , 4

#### Diagnosis.

Differs from *Solanum inornatum* Witasek in its elliptic leaves with attenuate bases and cuspidate apices, its leaf pubescence of upright spreading trichomes denser along the veins, its 2-foliate sympodial units, and its strongly recurved calyx lobes at anthesis. Also differs from *Solanum bradei* Giacomin & Stehmann by its conspicuous, linear-lanceolate calyx lobes.

#### Type.

BRAZIL. Rio de Janeiro: Mun. Nova Friburgo. Reserva Ecológica de Macaé de Cima, trilha para o Vale dos xaxins. 27 Oct 1990 (fl), *A. Amorim, B.C. Kurtz & L. Sylvestre 276* (holotype: RB [RB–00413518]; isotype: BHCB).

#### Description.

Herbs to shrubs, woody at base, few-branched, rhizomatous, up to 50 cm high, the branches on new growth ascending, becoming prostrate. Stems densely hispid-pubescent with uniseriate simple upright, spreading trichomes ca. 2.6 mm long, with up to 3 cells. Bark of older stems becoming light brown, glabrescent; new growth greenish brown, shiny. Sympodial units 2–foliate, not geminate. Leaves simple, solitary, the blades 3–11 × 1–5 cm, elliptic, chartaceous, slightly discolorous, drying darker above, not shiny, sparsely pubescent on both surfaces with simple trichomes like those of the stems, the trichomes denser along the veins (of any order); base attenuate, slightly decurrent onto the petiole; margins entire, ciliate, with spreading trichomes like those of the blade; apex acute to acuminate; petioles 6–12 mm long, with as pubescence like that of the stems; venation brochidodromous; midrib and secondary veins visible to the naked eye, prominent on both surfaces. Inflorescences sessile, lateral, unbranched cymes of 1–4 flowers; pedicels 4–12 mm long in flower, unknown in fruit, nearly contiguous. Calyx 3–5 mm long, deeply lobed, the tube ca. 1 mm long, the lobes 3–4 mm long, ca. 1mm wide, linear-lanceolate, densely pubescent abaxially with trichomes like those of the stem, glabrous adaxially, strongly recurved at anthesis. Corolla 1.4–1.8 cm in diameter, white, stellate, membranaceous, the lobes 5–7 × 2–3 mm, ovate-lanceolate, sparsely pubescent abaxially mainly along the midrib with trichomes like those of the calyx but shorter, up to 1.5 mm long, glabrous adaxially. Stamens 3–5 mm long, equal in length, the filaments ca. 1 mm long; anthers 2–4 mm long, ca. 1 mm wide, oblong, slightly connivent, yellow, the base cordate, the apex emarginate and poricidal, the subapical pores directed introrsely, not opening into longitudinal slits. Ovary glabrous; style white, 4–5 mm long, straight, cylindrical, the stigma light green, capitate. Fruit unknown.

**Figure 4.**
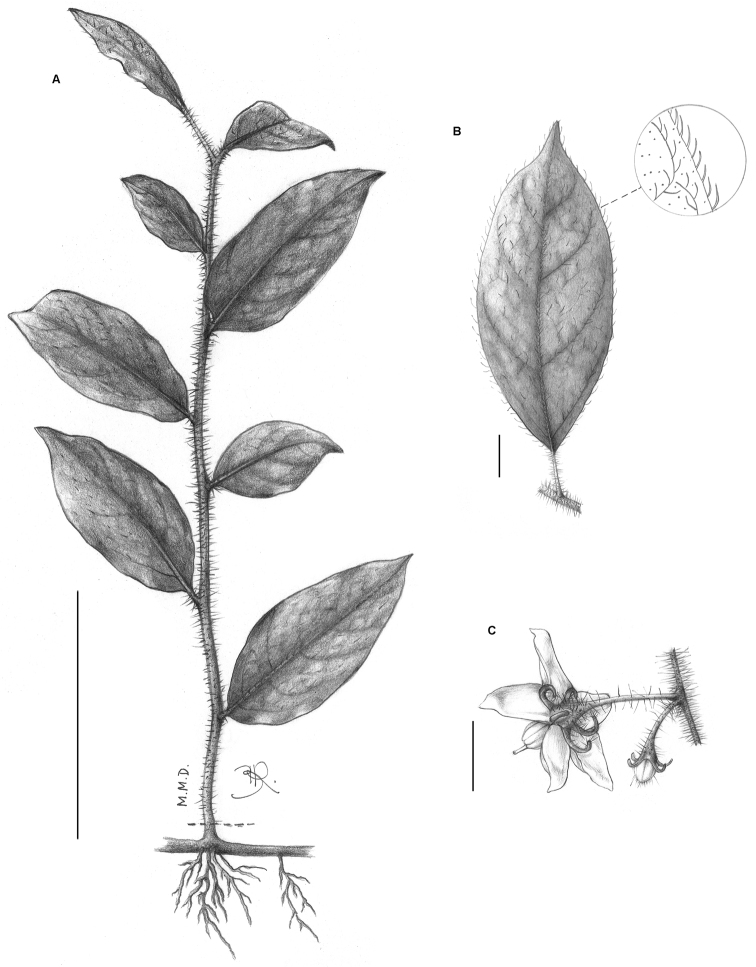
*Solanum friburgense* Giacomin & Stehmann. **A** Rhizomatous habit **B** Detail of the typical leaf shape and indumentum **C** Inflorescence detail, showing the diagnostic recurved calyx at anthesis. All from *Giacomin 940* (BHCB). Scale bars **A**= 10 cm; **B** = 1 cm; **C** = 5 mm. Drawings by M.M. Duarte and B. Raddichi.

#### Distribution.

Narrowly endemic, known from only two localities in the municipality of Nova Friburgo in the state of Rio de Janeiro, Brazil (Fig. 3). Both localities are within conservation units, one public (Reserva Ecológica de Macaé de Cima) and one private (RPPN Bacchus).

#### Ecology.

Rare in the understory of well-preserved fragments of the Brazilian Atlantic coastal rainforest, at elevations of about 1,500 m. The species always shows a well-developed rhizome system. This suggests the plant invests heavily in vegetative propagation, which is consistent with the few flowering specimens found in the field. In cultivation, so few flowers were produced that no crossing studies were performed.

#### Phenology.

The flowering material studied was collected in October and November but one of these is from cultivation. The fruits are unknown.

#### Etymology.

The species is named after the municipality where all known collections are from, Nova Friburgo, in the state of Rio de Janeiro.

#### Preliminary conservation status 

**(IUCN, 2013).** Critically Endangered (CR) B1; B2 ab (iii, iv). *Solanum friburgense* is known from two localities closely situated to each other within the same municipality, that represents an AOO of 8 km^2^. The type specimen was collected in 1990 and despite an intensive inventory that was recently carried out in the area (Lima and Guedes-Bruni 1997), no additional collections were made until 2009. Although the EOO could not be calculated because only two points are available, if the species is actually endemic to Nova Friburgo and surroundings it would probably fit the Critically Endangered category (less than 100 km^2 ^). Even considering the occurrence within two conservation units and the paucity of material available for analysis, we provisionally suggest it to be assessed as Critically Endangered, regarding it seems to have a reproductive system based on vegetative propagation (see Discussion below). Within RPPN Bacchus two large populations are known (about 50 individuals), but no flowering specimens were observed in the field over three consecutive years (2009, 2010 and 2011).

#### Discussion.

*Solanum friburgense* is the only species of the *Solanum inornatum* group that has 2-foliate non-geminate sympodial units. It shares the linear-lanceolate calyx lobes with *Solanum inornatum* but can be distinguished from it by having the calyx lobes strongly recurved at anthesis, and the leaf pubescence concentrated along the veins and only sparsely present on the mesophyll of the leaf lamina, while *Solanum inornatum* has straight to slightly curved calyx lobes and trichomes evenly distributed on veins and mesophyll. The leaf shape is also a good character to separate the species: *Solanum friburgense* has elliptic leaves with attenuate bases and cuspidate apices and *Solanum inornatum* ovate leaves with rounded bases and attenuate apices. *Solanum friburgense* can be readily distinguished from *Solanum bradei* in its conspicuous linear-lanceolate calyx lobes versus tiny deltate calyx lobes of the former.

As for all species of the *Solanum inornatum* group, *Solanum friburgense* has very discrete and almost hidden inflorescences, that are covered by the leaves if seen from above. This cryptic flowering could be the reason why the oldest known collection of the species is very recent, from the 1990s.

The species has an intriguing reproductive system, apparently based mainly on vegetative propagation. Although two large populations were found at RPPN Bacchus, no flowering specimens were seen in three consecutive years of field work encompassing almost three months of the rainy season, when the two only flowering specimens were collected. No fruit was seen in the field or produced in cultivation, and we believe that the few known specimens are the result of a very restricted distribution and not a lack of collecting effort. *Solanum friburgense* inhabits the understory of primary cloud forest fragments, which are not uncommon in the mountain ranges surrounding Nova Friburgo, suggesting that its restricted distribution is due to its vegetative reproductive strategy and not habitat specificity. Future efforts should be made to locate this species at nearby reserves in Rio de Janeiro state such as Parque Estadual dos Três Picos, in Nova Friburgo and Parque Nacional da Serra dos Orgãos, in Teresópolis, in order to search for additional populations.

#### Specimens examined.

**BRASIL. Rio de Janeiro:** **Mun. Nova Friburgo**. Macaé de Cima, Sítio do Srs. David e Isabel Muller, Trilha que leva para topo da serra, passando pela antena; espécime floresceu em casa de vegetação na Fundação Zoo-Botânica de Belo Horizonte, 1577 m, 22°22'24.64", 42°30'17.5", 20 Oct 2009 (fl), *L.L. Giacomin 940* (BHCB); RPPN Bacchus, Macaé da Cima, owned by David and Isabel Miller, Trilha do Telefone, 1555 m, 22°22'27"S, 42°30'04"W, 29 Apr 2010 (veg), *M.F. Agra et al. 7293* (BHCB, JPB).

### Solanum kriegeri

Giacomin & Stehmann sp. nov.

urn:lsid:ipni.org:names:77139691-1

http://species-id.net/wiki/Solanum_kriegeri

Figs 2D , 5

#### Diagnosis.

Differs from *Solanum bradei* Giacomin & Stehmann in its small shrub-like habit, its shiny leaves with extremely sparse pubescence, its sessile to subsessile inflorescences sometimes with a very short peduncle of up to 2 mm, and by its larger opaque fruit with more numerous seeds. Also differs from *Solanum inornatum* Witasek by having deltate, up to 2 mm calyx lobes.

#### Type.

BRAZIL**.** Minas Gerais: Parque Estadual da Serra do Ibitipoca, Proximidades da Lombada, 1650 m, 21°41'S, 43°53'W, 20 Jan 2005 (fl, fr), *R.C. Forzza, L.C. Assis, L.M. Bezerra, M.F. Calió & L.G. Temponi 3959* (holotype: RB; isotypes: BHCB, BM).

#### Description.

Herbs to small shrubs up to 50 cm tall, woody at base, often with a single stem or few branches, these primarily erect, ascending, becoming arched and pendant. Stems sparsely to moderately pubescent with simple uniseriate trichomes up to 1 mm long, with 2–3 cells, normally curved and antrorse, rarely spreading, sometimes geniculate. Bark of older stems becoming whitish, exfoliating, almost completely glabrous, that of new growth greenish brown. Sympodial units plurifoliate, normally not geminate, but if so, with leaves differing in size and shape. Leaves simple, 1.5–7 × 0.5–2 cm, narrowly elliptic, chartaceous to slightly coriaceous, concolorous, drying notably shiny on both surfaces, glabrous to glabrescent on both surfaces with sparse simple trichomes up to 0.6 mm long, with up to 2 cells, these most common along the midrib; base attenuate, not decurrent onto petiole; margins entire, sparsely ciliate, with trichomes like those of the leaf veins, lying antrorsely parallel to the margin; apex acute; petioles 2–5 mm long, with pubescence similar to the stems; minor leaves, if present, 0.4–1.2 × 0.3–0.6 cm, elliptic-ovate to circular, the base rounded to obtuse, the apex rounded to acuminate, the petioles absent to 2 mm long; venation brochidodromous; midribs and secondary veins visible to the naked eye, the midrib prominent on both blade surfaces, the secondary veins prominent abaxially and impressed adaxially. Inflorescences sessile to subsessile, lateral or subopposite the leaves, unbranched cymes with 1–6 flowers, the axis with the same pubescence as that of the stems; peduncle 1–2 mm long; rachis normally absent or rarely up to 6 mm long; pedicels 4–6 mm long in flower, 6–12 mm in fruit, articulated at the base, spaced up to 2 mm apart. Calyx to 5 mm long, the lobes 1–2 mm long in flower, up to 4 mm long in fruit, ca. 1 mm wide, deltate, glabrous to glabrescent abaxially, with trichomes if present like those of the stems, adaxially densely pubescent with capitate glandular trichomes less than 1 mm long, with single-celled stalks and a multicellular head; calyx not accrescent in fruit. Corolla 6–10 mm in diameter, white, stellate, membranaceous, the lobes 3–5 × 2–3 mm, ovate-lanceolate, glabrous on both surfaces. Stamens 2–3 mm long, equal in length, the filaments ca. 1mm long; anthers 1–2 mm long, ca. 1mm wide, oblong, slightly connivent, yellow, the base rounded, the apex emarginate and poricidal, the subapical pores directed introrsely, not opening into longitudinal slits. Ovary glabrous; style white, 3–5 mm long, straight, cylindrical, the stigma light yellow to greenish, capitate. Fruit a globose berry 6–12 mm in diameter, dull green when ripe, drying dark, glabrous. Seeds 6–12 per fruit, 3–5 × 2–3 mm, slightly swollen, reniform, with a small hollow at hilum region; the seed surface undulate, the margins flattened.

**Figure 5.**
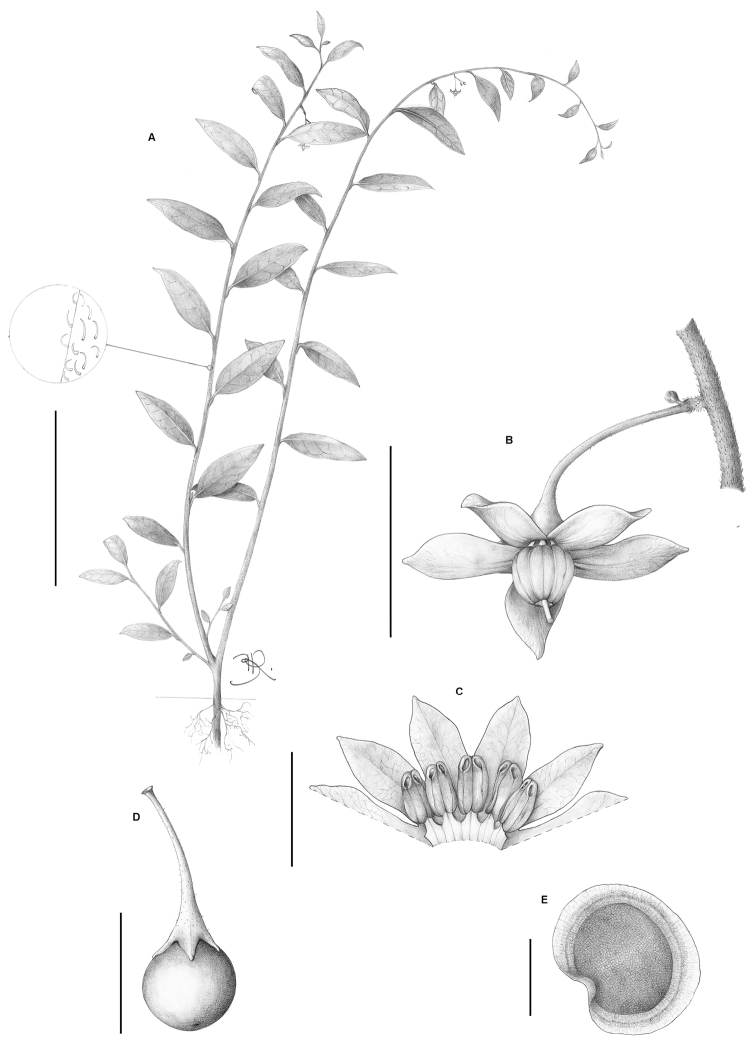
*Solanum kriegeri* Giacomin & Stehmann. **A** Habit with detail of the typical indumentum **B** Subsessile inflorescence detail showing the very short peduncle **C** Corolla cross section showing stamens **D** Fruit with small calyx lobes **E** Seed. All from *Giacomin et al. 770* (BHCB). Scale bars **A**= 10 cm; **B** and **D** = 1 cm; **C** = 5 mm; **E** = 2 mm. Drawings by B. Raddichi.

#### Distribution.

Endemic to Brazil in southern Minas Gerais state, close to the border with Rio de Janeiro State, where it is known from two adjacent mountain ranges within the Mantiqueira region, Serra do Ibitipoca and Serra Negra (Fig. 3). All known collections are from two conservation units, APA Serra da Mantiqueira and Parque Estadual do Ibitipoca.

#### Ecology.

Occasional to rare in the understory of well preserved dwarf cloud forests (*Floresta Ombrófila Densa Altomontana*; Veloso et al. 1991) and normally associated with sandy soils or quartzite outcrops, in elevations of about 1,500 to 1,900 meters above sea level. Although few flowers were produced in cultivation, crossing studies suggested this species is self-incompatible, like *Solanum bradei*.

#### Phenology.

Fertile specimens were collected between September and March. A flowering peak was observed between November and January and fully developed fruits were observed in January and March.

#### Etymology.

The epithet honors the Father Leopoldo Krieger, a Brazilian naturalist and founder of the CESJ herbarium (Juiz de Fora, Brazil), one of the most important collections in Minas Gerais state. In 1969, Dr. Krieger was hired as a Professor at Universidade Federal de Juiz de Fora, when he started collecting in the surrounding areas. His efforts at Serra do Ibitipoca led to the first collections of *Solanum kriegeri*. In herbarium sheets and in most databases, is common to find Krieger’s collections wrongly cited as “P.L. Krieger”; the “P” corresponds to the Portuguese word for Father (*Padre*) and not to a forename. Nevertheless we decided here to maintain the orthography used on individual labels in the cited material, noting with square brackets the common misuse of the “P.”.

#### Preliminary conservation status

**(IUCN, 2013).** Endangered (EN) B2 ab (iii, iv). The species is known from two localities that are about 30 km away from each other with six points available. The calculated EOO was of 34.3 km^2^ what would led to the Critically Endangered category while the AOO of 20 km^2^ led to Endangered. We have chosen here to assign it to Endangered, a less severe category for three reasons: the species occurs in more than one location, it is known from within a effectively protected area (Parque Estadual do Ibitipoca), and the other location where it is found is somewhat remote. Nevertheless, *Solanum kriegeri* is from a very specific habitat in well-preserved forest fragments and monitoring its populations is strongly recommended. In light of the deforestation pressure surrounding the areas where it is found, we surmise that it might be restricted to its few known localities.

#### Discussion.

*Solanum kriegeri* is most similar to *Solanum bradei*; both have small, deltate calyx lobes (1–2 mm long). It is, however, a much smaller plant than *Solanum bradei* with glabrous or glabrescent leaves, with sparse trichomes normally restricted to the midrib and a sessile to subsessile inflorescence. *Solanum kriegeri* occasionally has geminate leaves, but not as frequently as in *Solanum bradei* and, when present, the minor leaves are very reduced and look like stipules. The fruits of *Solanum kriegeri* are also distinctive; they are larger than those of *Solanum bradei* (6–12 versus 4–7.8 mm) due to the seed size and number (6–12 versus 2–4 seeds per fruit respectively). Another good field character is the fruit aspect at maturity: dull green in *Solanum kriegeri* and shiny, and translucent (watery) in *Solanum bradei*. Besides the calyx morphology *Solanum kriegeri* can be readily distinguished from *Solanum inornatum* and *Solanum friburgense* by the glabrescent indument of its shiny chartaceous leaves, the only species of the group that presents such feature.

*Solanum kriegeri* inhabits a very specific vegetation type and was thought to be endemic to Serra do Ibitipoca until recently when it was found in a neighboring mountain range. It is associated with dwarf cloud forests that grow as islands in highland grassy areas on sandy soils, normally within quartzite matrices. These formations are normally not as shaded as the habitats in which other species of the group are found, and although the soil is more well-drained, the atmospheric humidity is quite similar. It is common to find *Solanum kriegeri* growing in *Sphagnum* L. (Sphagnaceae) mats in these environments.

#### Specimens examined.

**BRAZIL**. **Minas Gerais:** **Mun. Lima Duarte.** Parque Estadual do Ibitipoca, Lombada, 8 Mar 2006 (fl, fr), *F.M. Ferreira et al. 1009* (CESJ); Parque Estadual da Serra do Ibitipoca, Mata próxima a Lagoa Seca, 1870 m, 21°40'S, 43°52'W, 24 Nov 2004 (fl), *R.C. Forzza et al. 3710* (RB); Parque Estadual do Ibitipoca, Mata e campo ao lado do alojamento, 21 Nov 2006 (fl, fr), *R.C. Forzza et al. 4322* (RB; BHCB); Conceição de Ibitipoca, Parque Florestal Estadual de Ibitipoca, Na mata, 29 Nov 1970 (fl), *[P.]L. Krieger & C.C. Urbano 9355* (CESJ, RB); Serra de Ibitipoca, perto de mata de galeria, 1500 m, 2 Nov 1973 (fl), *L. Krieger 13179* (CESJ); Parque Estadual do Ibitipoca, Na trilha de subida para a Lombada, 1623 m, 21°41'1"S, 43°52'24"W, 17 Mar 2009 (fl, fr), *L.L. Giacomin et al. 770* (BHCB); Parque Estadual do Ibitipoca, Mata nebular entre Lombada e Lagoa Seca, 1650 m, 21°40'57"S, 43°52'34"W, 27 Oct 2004 (fl), *B.R. Silva et al. 1369* (RB); Parque Estadual do Ibitipoca, Mata pluvial montana, 12 Nov 1987 (fl), *H.C. Sousa s.n*. (BHCB 14650; RB). **Mun. Rio Preto**. Serra Negra, Burro de Ouro, 1525 m, 21°58'09"S, 43°53'13"W, 17 Oct 2011 (fl), *L.L. Giacomin et al. 1642* (BHCB, BM, RB, UT); Serra Negra, Fragmento de floresta ombrófila altomontana anexa ao Pico das Três Divisas, 1601 m, 21°57'54"S, 43°52'57"W, 17 Oct 2011 (fl), *L.L. Giacomin et al. 1643* (BHCB, BM, UT).

##### Key to the species of the *Solanum inornatum* group

**Table:** 

1	Herbs to shrubs, up to 1.8 m; stems glabrescent to densely pubescent, if pubescent most of the trichomes antrorse or apressed; calyx lobes deltate, up to 2 mm long, not accrescent in fruit	2
–	Herbs to small shrubs, up to 50 cm; stems hirsute, the trichomes spreading to patent (sometimes hispid) or unordered (pointing in several directions, never completely antrorse); calyx lobes linear-lanceolate, 3-5 mm long, accrescent in fruit	3
2	Leaves matte when dried, conspicuously pubescent; inflorescences always pedunculate, peduncles 2.2–10 mm long; abaxial calyx surface conspicuously pubescent; fruits translucent green at maturity, 4–7.8 mm in diameter	*Solanum bradei* Giacomin & Stehmann
–	Leaves shiny when dried, glabrescent, with sparse trichomes mainly along midrib; inflorescences sessile to subsessile, with peduncles up to 2 mm long; abaxial calyx surface glabrescent; fruits dull green at maturity, 6–12 mm in diameter	*Solanum kriegeri* Giacomin & Stehmann
3	Sympodial units 2-foliate; leaves elliptic with attenuate bases and cuspidate apices; leaves with trichomes concentrated on veins of any order; calyx lobes strongly recurved at anthesis	*Solanum friburgense* Giacomin & Stehmann
–	Sympodial units 3-plurifoliate; leaves ovate-elliptic with rounded to subcordate bases and attenuate apices; trichomes evenly distributed on veins and mesophyll; calyx lobes straight, not strongly recurved at anthesis	*Solanum inornatum* Witasek

## Supplementary Material

XML Treatment for Solanum bradei

XML Treatment for Solanum friburgense

XML Treatment for Solanum kriegeri
